# Differentiating Blood, Lymph, and Primo Vessels by Residual Time Characteristic of Fluorescent Nanoparticles in a Tumor Model

**DOI:** 10.1155/2013/632056

**Published:** 2013-04-09

**Authors:** Sungwoo Lee, Jaekwan Lim, Jinmyung Cha, Jin-Kyu Lee, Yeon Hee Ryu, SungChul Kim, Kwang-Sup Soh

**Affiliations:** ^1^Nano Primo Research Center, Advanced Institutes of Convergence Technology, Seoul National University, Suwon 443-270, Republic of Korea; ^2^Department of Acupuncture, Korea Institute of Oriental Medicine, Daejeon 305-811, Republic of Korea; ^3^Materials Chemistry Laboratory, Department of Chemistry, Seoul National University, Seoul 151-747, Republic of Korea; ^4^Department of Acupuncture and Moxibustion, Wonkwang University Hospital, Gwangju 503-310, Republic of Korea

## Abstract

Fluorescent nanoparticles (FNPs) which were injected into a tumor tissue flowed out through the blood and lymph vessels. The FNPs in blood vessels remained only in the order for few minutes while those in lymph vessels remained for a long time disappearing completely in 25 hours. We found a primo vessel inside a lymph vessel near a blood vessel, and FNPs remained in the primo vessel for longer than 25 hours. In addition, we examined in detail the residual time characteristics of lymph vessels because it could be useful in a future study of fluid dynamical comparison of the three conduits. These residual time characteristics of FNPs in the three kinds of vessels may have implications for the dynamics of nanoparticle drugs for cancer chemotherapy.

## 1. Introduction

Distribution and behavior of nanoparticles injected into the skin are interesting subjects of biophysics as well as medical science. For example, time characteristics of the behavior of particulates in the skin depend on their physical size. Particles over a few hundred nanometers will be trapped in the interstitial space for a long time [1]. In the case of polyethylene glycol (PEG) coated 37 nm quantum dot particles, 60% remained 24 hrs after injection [2], and in the case of PEG coated 40 nm nanoparticles, over 25% was left 2 days after injection [3]. The distribution and behavior of nanoparticles injected in the skin were carefully studied before [4].

However, a more influential factor may be the residual time characteristics of the nanoparticles in the tissues such as blood, lymph, and primo vessels. When nanoparticles are injected into the skin, they are mostly carried away to other parts of the body by the flow of circulatory systems. If nanosize drugs are administered into the skin, their delivery to the desired organs or tissues as well as to the unintended organs depends upon these circulatory systems. Particularly, the drug dynamics depends upon time characteristics of the residual time in target organ, which, in turn, depends on morphological and biochemical features. Therefore, it is desirable to study the residual time characteristics of the nanoparticles injected in the skin. It is particularly significant in the case of drug administration into cancer tissues residing in the skin. 

The above-mentioned questions belong to another significant area of biomedical physics, namely, imaging. Fluorescence imaging analysis is a proper tool to deal with such questions. In this study, we combined an imaging technique with fluorescent nanoparticles (FNPs) to investigate residual time characteristics of subcutaneously injected particles into a cancer tissue of a xenograft mouse.

We used PEG-coated nanoparticles as surrogates for uncharged, biologically inert nanoscale materials. The nanoparticles were coated with silica containing rhodamine isothiocyanate (RITC) fluorescent dye and then finally coated with PEG [4]. The fluorescence of the dye inside the silica makes it possible to visualize with narrow wavelength light. 

Metastasis is one of the main causes of cancer mortality. Recently an additional path of metastasis besides the well-known blood and lymph paths was found and got much attention in the International Symposium of Primo Vascular System [5]. The primo vascular system (PVS) on tumor tissue was first observed in mice with subcutaneous xenografts of human lung cancer cell line NCI-H460 [6] and with intraperitoneally inoculated with the same cell line [7] with human lung cancer cell line NCI-H460. Metastasis through the PVS was observed and looked even more severe than lymphatic metastasis in some cases [8]. Observation of the PVS that had developed on melanoma tissue of black mice has also been reported [9].

The PVS is an anatomical structure corresponding to the acupuncture meridians and the acupuncture points of traditional Chinese medicine [10]. The PVS was shown to have a circulatory function, and the role of the PVS as a new circulatory system has been extensively studied [11]. Morphological studies, which showed a bundle structure of the PVS, were performed with hematoxylin and eosin (H&E) staining [12] and various types of electron microscopy [13–15]. To demonstrate liquid flow via the PVS, Alcian blue was injected into an organ-surface primo node. The average flow speed was measured to be 0.3 mm/sec in a primo vessel on the surface of a rabbit intestine [15].

In this research, we studied the residual time characteristics of FNPs in blood, lymph, and primo vessels which emerged from a tumor tissue in the ventral skin near the navel of a mouse. Before the measurements, we hypothesized that FNPs would be remained for the longest time in the primo vessel because of the porous surface structure of primo vessel compared to the dense outermost membrane and flow pressure of the lymph and the blood. Indeed, the results supported our hypothesis. In addition, we present the detailed residual time characteristics of FNPs in a lymph vessel because the lymph vessel is similar to a primo vessel in size, transparency and thus requires careful examination to distinguish from a primo vessel. The present study showed that time characteristics alone can be a simple and practical criterion for the distinction.

## 2. Materials and Methods

### 2.1. Cell Culture

NCI-H460 human lung cancer cells were obtained from the Korean Cell Line Bank (Seoul, Republic of Korea). The cells were cultured in a RPMI-1640 medium (GIBCO, USA) supplemented with 1% penicillin-streptomycin and 10% fetal bovine serum (GIBCO, USA) in 95% air and 5% CO_2_ at 37°C.

### 2.2. Animal Preparation and Cancer Model

Female athymic nude mice (BALB-c-nu/nu, aged 5 weeks old, weighing 15–20 g, *n* = 10; DooYeol Biotech, Seoul, Republic of Korea) were used. The mice were housed in a constant temperature (26°C), 60% relative humidity, and 12-hour light/dark cycle environment. The animals had *ad libitum* access to food and water. The research involving animals was approved by the Institute of Laboratory Animal Resources of Seoul National University.

The mice were inoculated subcutaneously in the abdomen with 2 × 10^6^ NCI-H460 human lung cancer cells (in a 0.2 mL of RPMI-1640 medium) for tumor formation under the skin. Three to four weeks after the inoculation, the animals were anesthetized with Zoletil/Rompun intraperitoneally. Nanoparticles (0.2 mL) were injected into a tumor tissue with a syringe. Immediately after the injection, fluorescence signals from the lymph vessels were taken with a fluorescence microscope (MVX 10, Olympus, Japan) from the outside of the skin without any surgery. Fluorescence signals from the blood vessels were not detected because the blood vessels of the tumor tissues were deeply located. In order to study residual time characteristics of nanoparticles in blood vessels from the tumor, a small area of the animal's skin was incised to expose blood vessels at the hypodermis to observe the residues of FNPs in the blood vessels. For this purpose, we injected nanoparticles for the second time because the nanoparticles injected before incision had been already flowed away.

### 2.3. Fluorescent Nanoparticles

Biocompatible nanoparticles containing organic fluorescence dye, rhodamine B isothiocyanate (RITC, Sigma-Aldrich, USA), within a silica shell (50 nm size, MNP@SiO_2_(RITC)s), were used in this study. The synthesis of the nanoparticles was described in earlier works and we quote it here briefly [4, 16]. Presynthesized cobalt ferrite nanoparticles (average 9 nm in diameter) were added to an aqueous polyvinylpyrolidone (PVP, Sigma-Aldrich, USA) solution. The PVP-stabilized cobalt ferrite nanoparticles were separated by the addition of acetone and subsequent centrifugation. The precipitated particles were redispersed in ethanol. Trimethoxysilane (Gelest, USA) modified by RITC was prepared from 3-aminopropyltriethoxysilane (Gelest, USA) and RITC under nitrogen. The synthesized silane-modified dye solution was then mixed with tetraethoxysilane (TEOS, Gelest, USA) and injected into the PVP-stabilized cobalt ferrite ethanol solution. The solution was subsequently polymerized on the surface of PVP-stabilized cobalt ferrites by the addition of ammonia as a catalyst to form RITC-incorporated silica-coated nanoparticle, MNP@SiO_2_(RITC)s. 

### 2.4. Imaging and Analysis

A fluorescent microscope (MVX10, Olympus, Japan) and a monochrome CCD camera (DP30BW, Olympus, Japan) were used to take fluorescence images of the flow of FNPs via primo, blood, and lymph vessels. After the FNPs (0.2 mL, 10 mg/mL in phosphate buffered saline) had been injected into the tumor tissue with an insulin syringe (31G Ultra-fine II, BD, USA), fluorescence images were taken for 25 hours. All images were taken at the same magnification, and the exposure time of each image was recorded to determine the fluorescence intensity.

The temporal changes of the fluorescence intensities of the blood vessels were recorded as movie files. The Image J program (1.42q version, NIH, USA) was used to analyze the temporal change of the fluorescence intensities of lymph vessels. In each fluorescence image, 5 points on lymph vessels and 5 points on the background were selected. The measured light strengths depended upon exposure times. Fluorescence intensities were obtained by correcting the exposure time effects of each image because images were taken for different exposure times. The light strengths were assumed to be proportional to the exposure time for a given intensity. The background intensities were subtracted from the intensities of lymph vessels to remove the background tissue autofluorescence. The mean and the standard deviation of the area-averaged intensities were then calculated.

### 2.5. Histological Analysis

The lymph vessel that contained the primo vessel was taken and fixed in 10% neutral buffered formalin (NBF) for 12 hours at 4°C. The sample was washed with tab water, dehydrated in a graded ethanol series, clarified in xylene, and embedded in paraffin. The paraffin block was sectioned transversely with a microtome in 5 *μ*m thicknesses.

Sample slides were photographed with a fluorescence phase-contrast microscope (BX51, Olympus, Japan) and a CCD camera (Infinity 2, Lumenera, Canada) to observe the fluorescence signal from the FNPs in the primo vessel. After a fluorescence signal was observed, sample slides were stained with H&E according to the general staining protocol for morphological observation and photographed with the same imaging setup.

## 3. Results

**Figure 1:**
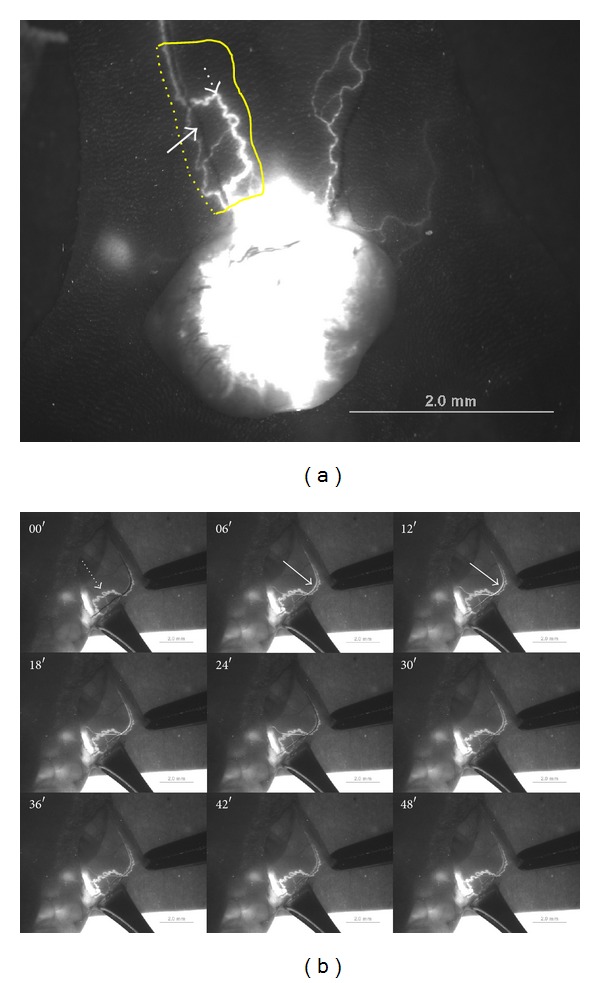
Flow of FNPs via blood vessel. (a) FNPs from the first injection flowed via a lymph vessel (dotted arrow) showed bright fluorescence signal that could be observed outside the skin. The FNPs had already flowed away in the blood vessel (*arrow*). The skin was incised along the dotted line and turned over. The opposite side of the skin in the box area was examined. (b) The panels are images captured from a movie taken immediately after the second injection of FNPs. At *t* = 0, the FNPs from the first injection were still in the lymph vessel (dotted arrow,the same one in (a) but seen from the inner side of the skin). FNPs were injected again at *t* = 0 and flowed via the blood vessel (arrows,the same one in (a)) rapidly, and the fluorescence signal was strong from 6 s to 18 s. FNPs had almost disappeared in a minute.

As shown in Figure 1(a), a bright fluorescence from the nanoparticles that had been injected into the tumor tissue of the subject nude mouse was observed with the fluorescent microscope. From the outside of the skin, one could see a bright fluorescence from nanoparticles flowing in the lymph vessel (broken arrow), but no signal from the blood vessel (arrow) was observed. Apparently, the lymph vessels were in a superficial layer of skin while the blood vessels were located more deeply. In order to observe the flow of nanoparticles in the blood vessel, we incised the skin along the dotted line in the figure and turned it over to examine its inner side.

In Figure 1(b), the panel at 00′ shows that the lymph vessel (broken arrow) was brightly seen from the inside and that the blood vessel was dark, without fluorescence from the nanoparticles. We injected FNPs for a second time into the tumor tissue, and the time 00′ means immediately after the injection. The panel at 06′ shows that the lymph vessel became brighter and that the blood vessel (arrow) became bright due to the flow of FNPs. The brightness of the lymph vessel persisted for more than 48 seconds after the injection. However, the blood vessel became nearly dark within one minute. This phenomenon in which FNPs flowed away quickly in the blood vessel within a few minutes while FNPs flowed and remained in the lymph vessel in the order for several hours was generally observed in the current work as well as previous other experiments [4].

**Figure 2:**
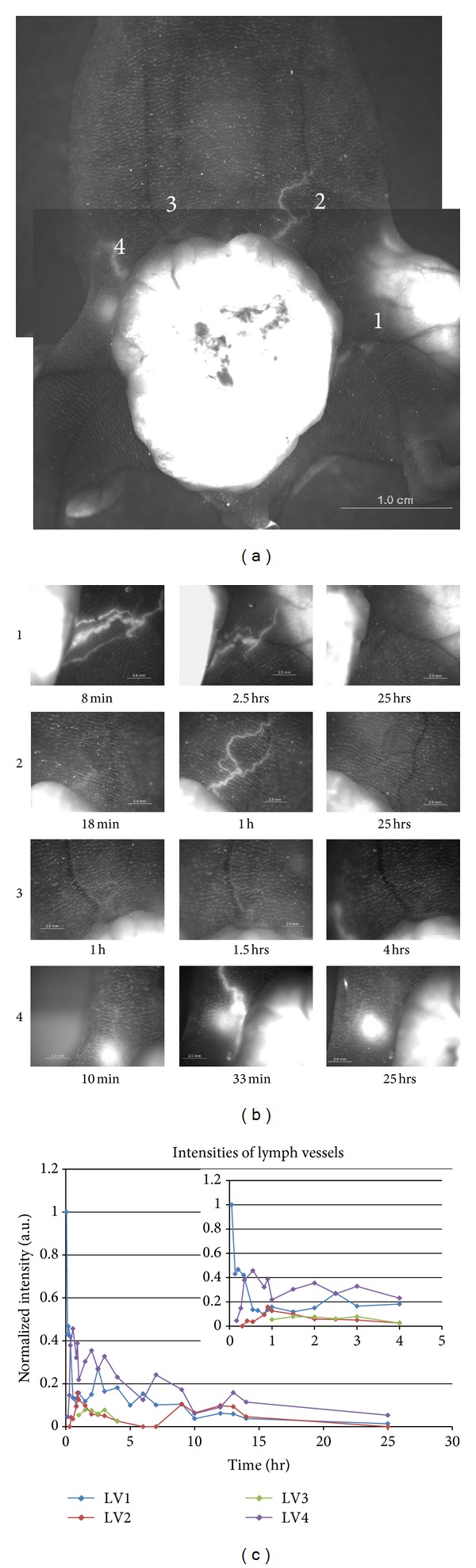
(a) Ventral view of the tumor and its lymph vessels. Four lymph vessels (indicated by numbers) were visualized with the flow of FNP and were detected without any surgery due to the strong fluorescence from the FNPs. (b) Selected fluorescence images of each lymph vessel. Images were taken for 25 hours in a time series. The first column shows the moment when a noticeable signal appeared in each of the four vessels, respectively. The second column, except lymph vessel 1 (LV1), represents the moments when the signals became maximum in each lymph vessel. The exceptional case LV1 had the strongest signal in the beginning, and a second peak occurred at 2.5 hours. The third column shows the time that the signal of each lymph vessel became almost invisible. (c) Temporal changes of the normalized fluorescence intensities of the four lymph vessels in (b). The maximum intensity of LV1 was fixed to be 1 (arbitrary units). More detailed fluorescence intensities from *t* = 0 to 4 hours are presented in the inset.

In Figure 2, we presented time characteristics of the flow of FNPs in four different lymph vessels from a tumor tissue. As shown in the panels of Figure 2(b) and in the graph of Figure 2(c), the times of the first appearances of the FNP signals for the four lymph vessels were different but were all within one hour from the time of injection. The maximum bright signal also occurred within one hour from start up. The FNP signal remained visible for up to 15 hours, but definitely became invisible within 25 hours.

**Figure 3:**
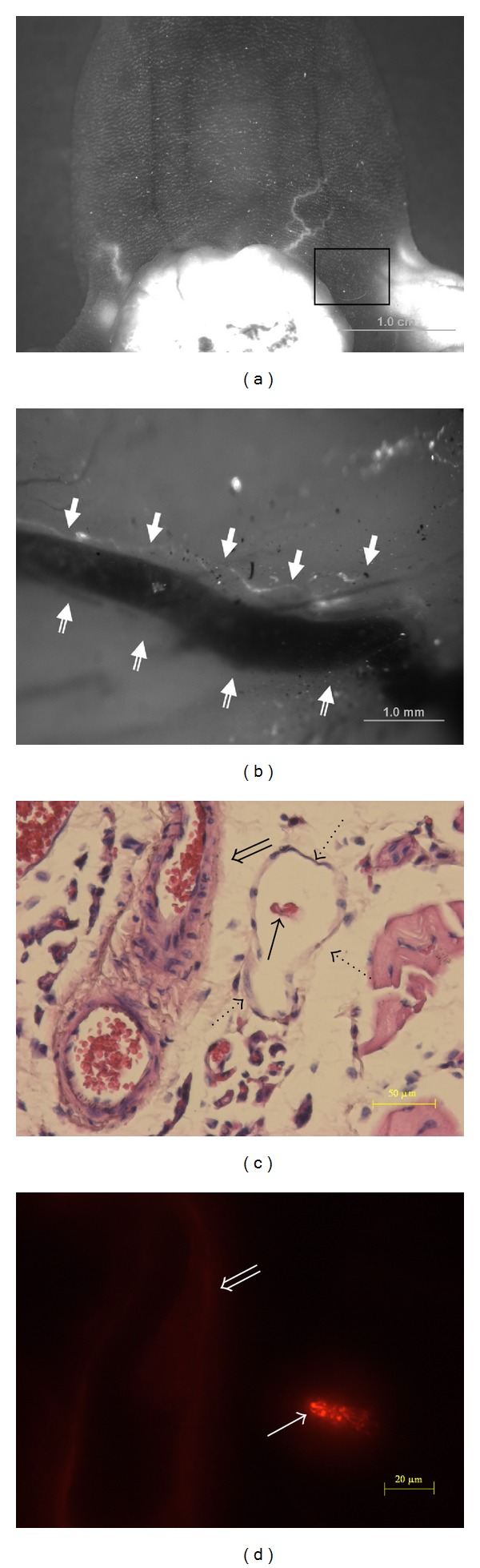
(a) Ventral view of the tumor after the injection of FNPs. The rectangular area was investigated after the skin had been incised and turned over. (b) Fluorescence image of the primo vessel (arrows) 25 hours after the injection of FNPs. The primo vessel appeared to be accompanying the blood vessel (double arrows) in the hypodermis. This image was taken after the skin in the rectangular area of (a) had been turned over. (c) H&E image of a cross section of the same sample as in (b). A primo vessel (arrow) accompanying the blood vessel (double arrow) was, in fact, located inside the lymph vessel (dotted arrows). (d) Fluorescence signal from the FNPs in the primo vessel (arrow) accompanying the blood vessel (double arrow). Notice that the FNPs remained only in the primo vessel but not in the lymph vessel at all. The weak signals at other sites are due to tissue autofluorescence. This image is a different slide from (c), but both are from the same specimen. (More detailed analysis on this figure was presented in [17].)

In Figure 3, a long-lasting fluorescence of FNPs was observed to accompany a blood vessel from a tumor tissue (Figure 3(a)). The blood vessel was in the hypodermis and was observed after the incised skin had been turned over. The blood vessel (double arrows) was about 0.8 mm thick, and the persistent FNP-bearing primo vessel (arrows) accompanying the blood vessel was very thin as shown in Figure 3(b). We examined a cross section of the blood vessel (double arrows) and its neighbors and found surprisingly that the FNP-bearing primo vessel (arrows) was inside a lymph vessel (dotted arrows) (Figure 3(c)). The thickness of the FNP-bearing primo vessel (arrow) was about 20 *μ*m (Figure 3(d)), which was a typical size of the primo vessels observed inside lymph vessels in earlier works [17–20]. This long remnant FNP signal is a residual time characteristic of a primo vessel, as more details of this phenomenon were reported earlier [21].

## 4. Discussion

We compared residual time of nanoparticles in blood, lymph, and primo vessels in an NCI-H460 xenocrafted tumor mouse. FNPs, instead of ordinary dyes such as Trypan blue, Alcian blue, and Janus green B which were used in PVS research, were used as a tracer [17, 20, 22–24]. FNPs can be detected without surgery and are suitable for observing the extremely small residues of nanoparticles in blood, lymph, and primo vessels. Fluorescence signals from the FNPs in blood, lymph, and primo vessels were observed with a fluorescent stereo microscope for a long time (up to 25 hours). The residual times of the FNPs showed marked differences.

Measuring the residual time of FNPs in blood vessels was difficult because the fluorescence signal from the FNPs in blood vessels could not be detected from outside the skin. Therefore, the skin was incised and turned over to expose blood vessels in hypodermis. Because the FNPs rapidly flowed away in a few minutes, the fluorescence signal disappeared likewise (Figure 1(b)). Due to the short residual time of the FNPs in the blood vessel, they were not sufficiently uptaken by the PVS in the blood vessels to reveal its presence even when there was one floating in the blood stream.

In addition, the flow characteristic of lymph vessels was analyzed. Four lymph vessels at the ventral side of a mouse were observed (Figure 2). We could observe a fluorescence signal from each lymph vessel from outside the skin. These four lymph vessels showed different residual time characteristics. The fluorescence signal from lymph vessel 1 (LV 1) decreased monotonically with increasing time. The fluorescence signal from LV 1 remained for up to 25 hours after the injection, but the signal had decreased substantially in 1 hour. Lymph vessel 2 (LV 2) showed an interesting phenomenon, three peaks of fluorescence intensity one each at 1 hour, 9 hours, and 12 hours after the injection. This recurring phenomenon might occur because of the internal dynamics of the tumor tissue, but the exact internal structure of the tumor tissue was not investigated. Lymph vessel 3 (LV 3) and lymph vessel 4 (LV 4) also showed recurring peaks. Further detailed analysis of the recurrence will be possible only when the interior structure of the tumor tissue is determined in future studies. The FNP signal in the lymph vessel disappeared within 25 hours after the injection.

It was remarkable that a primo vessel inside a lymph vessel was incidentally detected. The existence of a primo vessel floating inside a lymph vessel has been reported several times [17–20], but its presence in a lymph vessel connected to a tumor has rarely been observed [21]. Their existence was noticed due to the FNPs that persisted because they were captured by the abundant fibers in the primo vessel, which is a characteristic feature of a primo vessel compared with the blood or lymph vessels [12]. We note that PVS was closely related to cancer metastasis [8, 25] and a lymph vessel from a tumor that contains a PVS may play a significant role in cancer metastasis. As a concrete example, a breast cancer that metastasizes through the axillary lymph node one needs to detect the particular lymph vessel that contains a PVS in it. In such a case, it is critical to be able to distinguish the lymph vessel that contains a PVS from those which do not. The long residual time of the FNPs in a PVS will be useful to identify this particular lymph vessel. The current method is more advantageous than conventional histological methods which are very difficult to detect the PVS in a lymph vessel.

This study was limited in that the sensitivity of the fluorescence detection was not good enough to detect the signals from blood vessels in the hypodermis and from the primo vessel inside a lymph vessel. Due to this limitation, the flow of nanoparticles in the primo vessel was not directly observed, and therefore the nanoparticles might flow in the lymph flow and were simply absorbed by the primo vessel. In addition, because of the rareness, we could not detect more primo vessels in the lymph vessels and could not perform detailed histological analysis either. Detailed structure of the PVS in a lymph vessel was studied with the transmission electron microscopy [18], and immunohistological images of the PVS that were taken from lymph vessels were obtained with confocal laser scanning microscopy [26]. 

Finally, we consider a recent significant work on the relationship between the acupuncture meridian and the PVS [27]. The authors reported that “the PVs in surface of internal organs did not have an effect in regulating gastric motility. The PVs in the surface of internal organs were involved neither in the inhibition of the gastric motility induced by acupuncturing at CV12 nor in the facilitation of gastric motility induced by acupuncturing at ST36. Further research about the functional relationship between the PVs and meridian is needed in the future.” This investigation is very valuable for the functional aspects of the PVS. Its results are valid for the subclass of PVS on the surface of internal organs (OS-PVS). There is a complicated network of five subclasses of PVS, and the most important ones with respect to the intestinal motility are those along blood vessels and nerves as implied in Kim's work [10]. The OS-PVS is deeply related to stem cell like functions and immune functions [26]. It is timely that functional aspects of the PVS are to be studied with respect to both Western and Eastern medicines.

## 5. Conclusion

FNPs injected into tumor tissue had residual time of just a few minutes in blood vessels, of twenty hours in lymph vessels, and of beyond 25 hours in a primo vessel. These residual time characteristics in the three different kinds of vessels can be useful for distinguishing these vessels in the cases of in vivo imaging with nanoparticles. Nanosized drugs injected into tumor tissue flow through blood and lymphatic vessels but could stay for a long time in primo vessels. Our results can provide valuable basic information for drug administration in skin using nanoparticles, and in the case of cancer metastasis through lymph vessels.
